# Molecular dissection of resistance gene cluster and candidate gene identification of *Pl*_17_ and *Pl*_19_ in sunflower by whole-genome resequencing

**DOI:** 10.1038/s41598-019-50394-8

**Published:** 2019-10-18

**Authors:** Guojia Ma, Qijian Song, William R. Underwood, Zhiwei Zhang, Jason D. Fiedler, Xuehui Li, Lili Qi

**Affiliations:** 1 Department of Plant Sciences, North Dakota State University, Fargo, ND 58108 USA; 2 USDA-Agricultural Research Service, Soybean Genomics and Improvement Laboratory, Beltsville, MD 20705-2350 USA; 3 USDA-Agricultural Research Service, Edward T. Schafer Agricultural Research Center, Fargo, ND 58102-2765 USA; 4 Department of Agronomy, Inner Mongolia Agricultural University, Huhhot, Inner Mongolia, 010019 China

**Keywords:** Genetics, Plant sciences

## Abstract

Sunflower (*Helianthus annuus* L.) production is challenged by different biotic and abiotic stresses, among which downy mildew (DM) is a severe biotic stress that is detrimental to sunflower yield and quality in many sunflower-growing regions worldwide. Resistance against its infestation in sunflower is commonly regulated by single dominant genes. *Pl*_17_ and *Pl*_19_ are two broad-spectrum DM resistance genes that have been previously mapped to a gene cluster spanning a 3.2 Mb region at the upper end of sunflower chromosome 4. Using a whole-genome resequencing approach combined with a reference sequence-based chromosome walking strategy and high-density mapping populations, we narrowed down *Pl*_17_ to a 15-kb region flanked by SNP markers C4_5711524 and SPB0001. A prospective candidate gene HanXRQChr04g0095641 for *Pl*_17_ was identified, encoding a typical TNL resistance gene protein. *Pl*_19_ was delimited to a 35-kb region and was approximately 1 Mb away from *Pl*_17_, flanked by SNP markers C4_6676629 and C4_6711381. The only gene present within the delineated *Pl*_19_ locus in the reference genome, HanXRQChr04g0095951, was predicted to encode an RNA methyltransferase family protein. Six and eight SNP markers diagnostic for *Pl*_17_ and *Pl*_19_, respectively, were identified upon evaluation of 96 diverse sunflower lines, providing a very useful tool for marker-assisted selection in sunflower breeding programs.

## Introduction

Sunflower (*Helianthus annuus* L.) is native to North America and is an important economic crop worldwide because of its edible seeds (confectionary sunflower) and oil products (oilseed sunflower), as well as for ornamental purposes. Like other crops, sunflower production is often hindered by many factors, including biotic (e.g., diseases, pests, birds, etc.) and abiotic stresses (e.g., drought, waterlogging, and salinity). Among the diseases, downy mildew (DM), incited by the oomycete *Plasmopara halstedii* (Farl.) Berlese & de Toni, is one of the most severe biotic factors affecting sunflower production worldwide, particularly in Europe and North America^1,2^. Typical symptoms of DM infections include leaf chlorosis, seedling dwarfing, and white sporulation on the underside of leaves (https://www.ag.ndsu.edu/extensionentomology/recent-publications-main/publications/A-1331-sunflower-production-field-guide). Seedling death is commonly seen upon DM infection. Despite some survival, the infected plants display stunted growth and typically yield little or no seeds. Generally, it is publicly accepted that the deployment of disease resistance in crops is the most effective and environmentally sound means of controlling disease infestation. The identification of resistance is prerequisite for sunflower breeding against pathogen infection.

Resistance (*R*) against downy mildew in sunflower is, in most cases, governed by a single dominant gene (designated as *Pl*), although some partial and quantitative resistance have also been reported^3,4^. To date, a total of 36 *Pl* genes, *Pl*_1_*–Pl*_35_, and *Pl*_*Arg*_, have been reported from the DM resistance pool in cultivated sunflower and its wild relatives (Supplementary Table S1). However, DM resistance is often rendered ineffective by the rapid genetic changes in the pathogen populations due to the coevolution between the pathogen and sunflower host^5,6^. Some of the *Pl* genes that have been widely used to combat DM infection in sunflower have already been ineffective against new races of *P*. *halstedii*, such as *Pl*_6_ and *Pl*_7_^7–9^. A recent survey showed that only the *Pl*_*Arg*_, *Pl*_15_, *Pl*_17_, *Pl*_18_, and *Pl*_33_ genes remained effectively resistant against a total of 185 *P*. *halstedii* isolates collected from North Dakota, South Dakota and Nebraska sunflower production regions in the United States when a total of twelve known DM *R* genes were tested, including *Pl*_1_, *Pl*_2_, *Pl*_5_, *Pl*_6_, *Pl*_13_, *Pl*_15_*–Pl*_18_, *Pl*_21_, *Pl*_33_, and *Pl*_*Arg*_^10^.

Intensive breeding efforts in sunflower have narrowed down the genetic variability of the sunflower genome, resulting in a constant need to identify and deploy new agronomically important genes. There are 53 wild sunflower species belonging to the *Helianthus* genus, which are invaluable reservoirs of agronomically desirable genes^11–13^. The oil maintainer line HA 458 (PI 655009) is resistant to all North American *P*. *halstedii* races identified thus far^10,14^. It harbors the DM *R* gene, *Pl*_17_, originating from the wild *H*. *annuus* L. accession PI 468435. The other DM *R* gene, *Pl*_19_, was also identified from the wild *H*. *annuus* L. accession PI 435414^15^. Both *Pl*_17_ and *Pl*_19_ genes were previously mapped to sunflower chromosome 4 corresponding to linkage group 4 in a similar position^16,17^. Recently, four additional novel DM *R* genes, *Pl*_27_*–Pl*_29_ and *Pl*_33_, were identified in proximity to *Pl*_17_ and *Pl*_19_ on chromosome 4^18,19^, while *Pl*_17_, *Pl*_19_, and *Pl*_33_ were in an interval spanning a physical distance of approximately 3.2 Mb when the flanking markers were positioned on chromosome 4 pseudomolecules of the HA412-HO genome sequence^16,17,19^.

The three DM *R* genes, *Pl*_17_, *Pl*_19_, and *Pl*_33_, were highly effective toward the most predominant and virulent races of *P*. *halstedii* and have not been widely used for commercial sunflower production. The broad-spectrum DM resistance and similar position of these *R* genes make it infeasible to select individuals harboring respective *R* gene based on phenotyping. Diagnostic molecular markers would provide a timely and accurate selection tool for sunflower breeding programs and would be developed with the advancement of rapidly developing sequencing technology combined with the single nucleotide polymorphism (SNP) genotyping system.

The publicly available genomic resources of the two assembled and annotated genome reference sequences of HA412-HO and XRQ in sunflower are powerful tools to study the genetic basis of agronomically important traits, to utilize sequence information for marker development, and to dissect the trait-governing genes genetically and molecularly^20^. Whole-genome resequencing can be utilized for efficiently identifying SNP, insertion and deletion (InDel), structure variation (SV), and copy number variation (CNV) in a massively parallel manner. The extremely high distribution of SNPs in the genomes of all organism makes it a powerful genetic tool for population genetics studies and marker-trait association analyses. However, current use of PCR-based approaches for genotyping of individual SNPs of special interest is still limited by accuracy, throughput, simplicity, and operational costs. An innovative SNP genotyping method has been developed in our laboratory, which adapts to multiple platforms and throughputs, allowing a PCR-based technology to genotype individual SNPs^16,21,22^. In the current study, we report the use of reference sequence-based chromosome walking toward the target genes, *Pl*_17_ and *Pl*_19_, identify candidate genes, and develop user-friendly SNP markers diagnostic for *Pl*_17_ and *Pl*_19_.

## Results

### Saturation and fine mapping of Pl_17_

**Figure 1:**
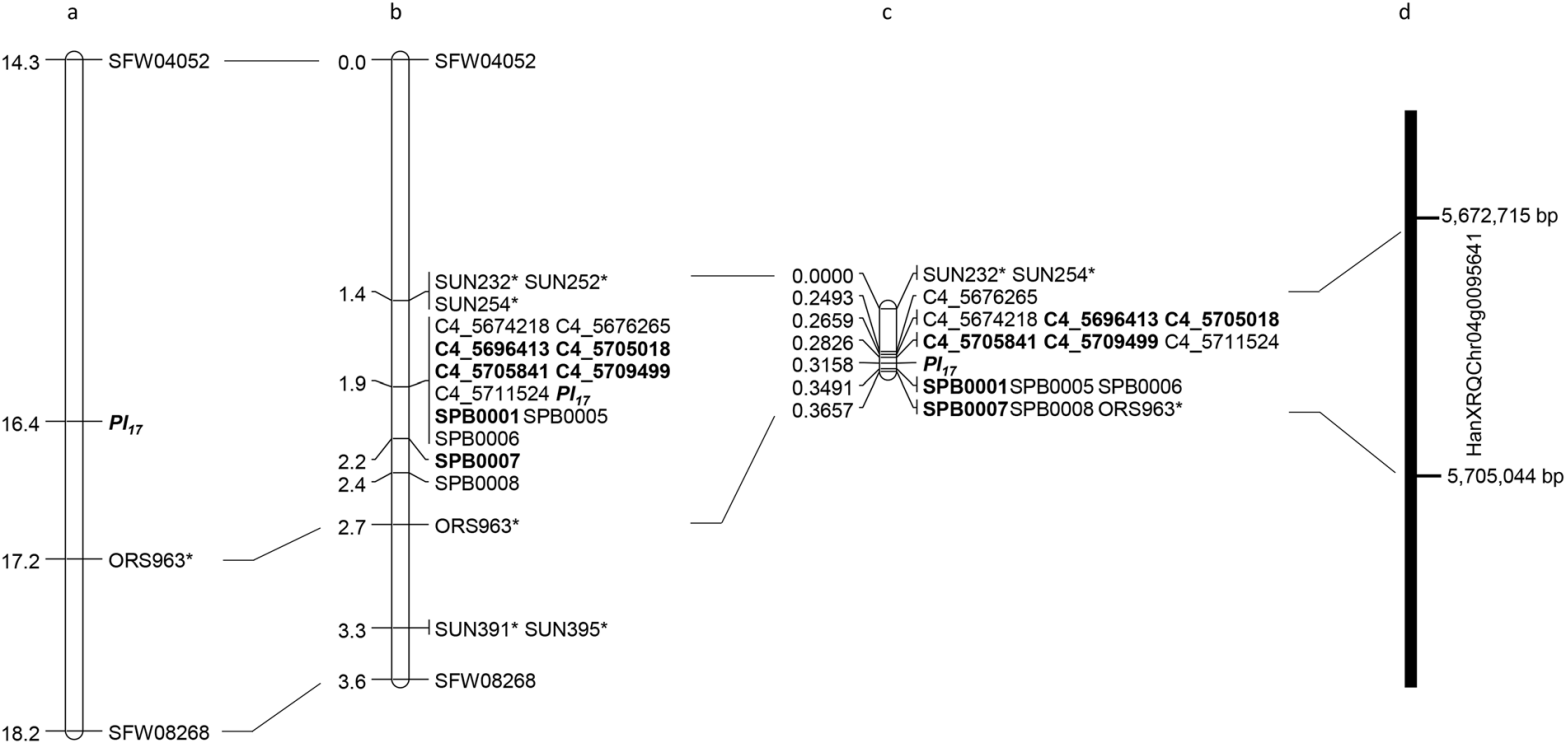
*Pl*_17_ genetic maps. (**a**) *Pl*_17_ basic map (Qi et al. 2015); (**b**) *Pl*_17_ saturation map; (**c**) *Pl*_17_ fine map; (**d**) physical position of the *Pl*_17_ candidate gene. The diagnostic marker for *Pl*_17_ is shown in bold. *SSR/STS markers.

Two strategies were adopted for marker development. At first, the genome sequence of chromosome 4 was extracted from the HA412-HO reference assembly from 3,621,089 to 6,852,749 bp and the XRQ assembly from 5,662,479 to 5,707,598 bp, which covers the *Pl*_17_ and *Pl*_19_ loci reported in previous studies^16,17^. A total of 101 pairs of primers, including 40 STSs and 61 SSRs were screened for polymorphisms between the parents HA 458 (*Pl*_17_) and HA 234. Polymorphic markers were further used to genotype 186 F_2_ individuals of HA 234 × HA 458. Five markers were mapped around the *Pl*_17_ locus (Table 1), reducing the *Pl*_17_ gene interval from 2.9 cM between SFW04052 and ORS963 to 1.3 cM between SUN232 and ORS963 (Fig. 1a,b).

**Table 1:** Primer sequences of mapped SSR and STS markers in *Pl*_17_ and *Pl*_19_ saturation maps.

Primer ID	Category	Motif	No. of Repeats	Sequence Resource	Forward Primer	Reverse Primer	Amplicon Size (bp)	Mapped to
SUN232	SSR	ga	16	HA412-HO	TGTTTGAAAGGGAGACCACA	GGCGAGTTTATTTTGGGTGA	234	*Pl*_17_ and *Pl*_19_
SUN252	STS	—	—	HA412-HO	AACGACATGCACATGGAAAA	AGAAAGCCTGCCAAACAAAA	233	*Pl*_17_
SUN254	STS	—	—	HA412-HO	GGACCATATGGGGTTTTCCT	TTCGGGCATATTTCAAGTCC	179	*Pl*_17_ and *Pl*_19_
SUN287	STS	—	—	HA412-HO	TGTGATTGAAAAACCGGTCA	TACGGGTCAAACGGGTAAAA	225	*Pl*_19_
SUN367	SSR	ag	12	HA412-HO	ATGGATGCCTTGCTCATCCC	CACTCCCATGCCCCTTACAG	251	*Pl*_19_
SUN375	SSR	ag	8	HA412-HO	AATGATGAGGATGGCCGCAG	GATCAACTCGAAACCGGCAC	239	*Pl*_19_
SUN391	SSR	gt	10	HA412-HO	AATCACGCGAGAGTGAGGTG	GACGAGTGTGTGTTAGGGCA	220	*Pl*_17_ and *Pl*_19_
SUN394	SSR	ct	5	HA412-HO	GGAATGGGCATACCTGTGGG	TGGCTTGACCCGTTTTCACA	265	*Pl*_19_
SUN395	SSR	tg	5	HA412-HO	TGTACAGCCCCAAATACCGC	ATCCGGATTCGAGCCATTACC	268	*Pl*_17_
SUN444	SSR	ac	5	XRQ	CGAACAAACACACACACATTG	TGCATGTACAGCCCCAAATA	154	*Pl*_19_
SUN446	SSR	ca	10	XRQ	TCATGACGAGAGAGACGAGTG	CAAACTTTTTGCTTCGCACA	151	*Pl*_19_
SUN458	SSR	tg	7	XRQ	CAGGCATTTTGTGTGTGGTT	CCACAAAAATTCAGGCAAAAG	198	*Pl*_19_
SUN461	SSR	gaa	5	XRQ	AGTAATGGCCAACATCATGC	GGATGGCCCTAAAAACCATT	208	*Pl*_19_

To further narrow down the *Pl*_17_ gene region, a total of 80 SNPs was identified from the HA458-WGS1 (27 SNPs) and HA458-WGS2 (53 SNPs) in the targeted gene interval. Ten contigs were identified from the HA458-WGS1, which fell in the *Pl*_17_ interval and were used as queries to align against the HA412-HO and XRQ references with BLASTn. A total of 27 SNP markers (SPB0001*–*SPB0027) were selected between the HA458-WGS1 and the XRQ sequence (Supplementary Table S2). Another 53 SNP markers were selected based on SNPs/InDels between the HA458-WGS2 and the two references, with 16 from the 69.3 kb segment (5,951,824*–*6,021,131 bp) of the HA412-HO assembly and 37 from the 138.1 kb segment (5,600,607*–*5,738,736 bp) of the XRQ assembly. Of 80 SNP markers screened, 12 showed polymorphisms between HA 234 and HA 458 and were used to genotype the F_2_ population. Linkage analysis indicated that ten SNP markers co-segregated with *Pl*_17_ and one (SPB0007) was proximal to *Pl*_17_ at a 0.3 cM genetic distance (Fig. 1b).

Fine mapping of *Pl*_17_ was performed to dissect the SNP marker cluster co-segregating with *Pl*_17_ and to increase the map resolution. The two previously reported *Pl*_17_ flanking markers, SNP marker SFW04052 and SSR marker ORS963 covering a 2.9 cM interval (Fig. 1a), were used to genotype the 3,008 F_3_ individuals from the selected F_3_ families that were heterozygous for *Pl*_17_. One hundred and three recombinants were identified and advanced into the next generation. The SSR marker SUN232 identified from saturation mapping was closer to the *Pl*_17_ locus than SFW04052 and was then used to screen the 103 recombinants (Fig. 1b). Twenty-two of them were found to have recombination events in the target interval of 1.3 cM flanked by the SSR markers SUN232 (0.5 cM) and ORS963 (0.8 cM), and their advanced generation was inoculated with *P*. *halstedii* race 734 for the resistance test.

The 12 polymorphic SNP markers mapped to the *Pl*_17_ interval between markers SUN232 and ORS963 using the 186 F_2_ individuals were further used to genotype 22 recombinants identified from 3,008 F_3_ individuals. As a result, *Pl*_17_ was placed in a 0.0665 cM interval at the upper end of chromosome 4, flanked by markers C4_5711524 (0.0332 cM) and SPB0001 (0.0333 cM) (Fig. 1c). Most of the markers were physically in accordance with their genetic positions, although five SPB SNPs had a reversed order in both the HA412-HO and XRQ assemblies compared with their genetic positions (Table 2). The flanking markers C4_5711524 and SPB0001 delimited *Pl*_17_ to a 15 kb interval on the XRQ genome assembly.

**Table 2:** Genetic and physical positions of markers linked to *Pl*_17_ on the fine map of sunflower chromosome 4.

Marker	No. recombination	Genetic distance (cM)	Physical position on XRQ assembly (bp)	Physical position on HA412-HO assembly (bp)
SUN232^†^		0	NA	5,580,196-5,580,326
SUN254^†^	0	0	5,331,535-5,331,713	5,593,058-5,593,237
C4_5676265	15	0.24933	5,676,065-5,676,465	6,076,762-6,077,162
C4_5674218	1	0.26595	5,674,018-5,674,418	6,078,809-6,079,209
**C4_5696413**	0	0.26595	5,696,213-5,696,613	6,154,336-6,154,735
**C4_5705018**	0	0.26595	5,704,818-5,705,218	6,028,462–6,028,862
**C4_5705841**	1	0.28257	5,705,641–5,706,041	6,027,639–6,028,039
**C4_5709499**	0	0.28257	5,709,299–5,709,699	6,021,349–6,021,749
C4_5711524	0	0.28257	5,711,324–5,711,724	6,627,884–6,628,284
***Pl***_**17**_	2	0.31581	—	—
**SPB0001**	2	0.34905	5,696,076–5,696,181	5,950,918–5,951,024
SPB0005	0	0.34905	5,704,420–5,704,545	5,947,019–5,947,144
SPB0006	0	0.34905	5,703,949–5,704,083	5,947,481–5,947,612
**SPB0007**	1	0.36567	5,703,815–5,703,944	6,152,126–6,152,255
SPB0008	0	0.36567	5,703,815–5,703,944	6,152,126–6,152,255
ORS963^†^	0	0.36567	6,676,888–6,676,910	6,970,802–6,971,141

^†^SSR/STS markers. The diagnostic SNP marker for *Pl*_*17*_ is shown in bold. NA, not available.

### Saturation and fine mapping of Pl_19_

**Figure 2:**
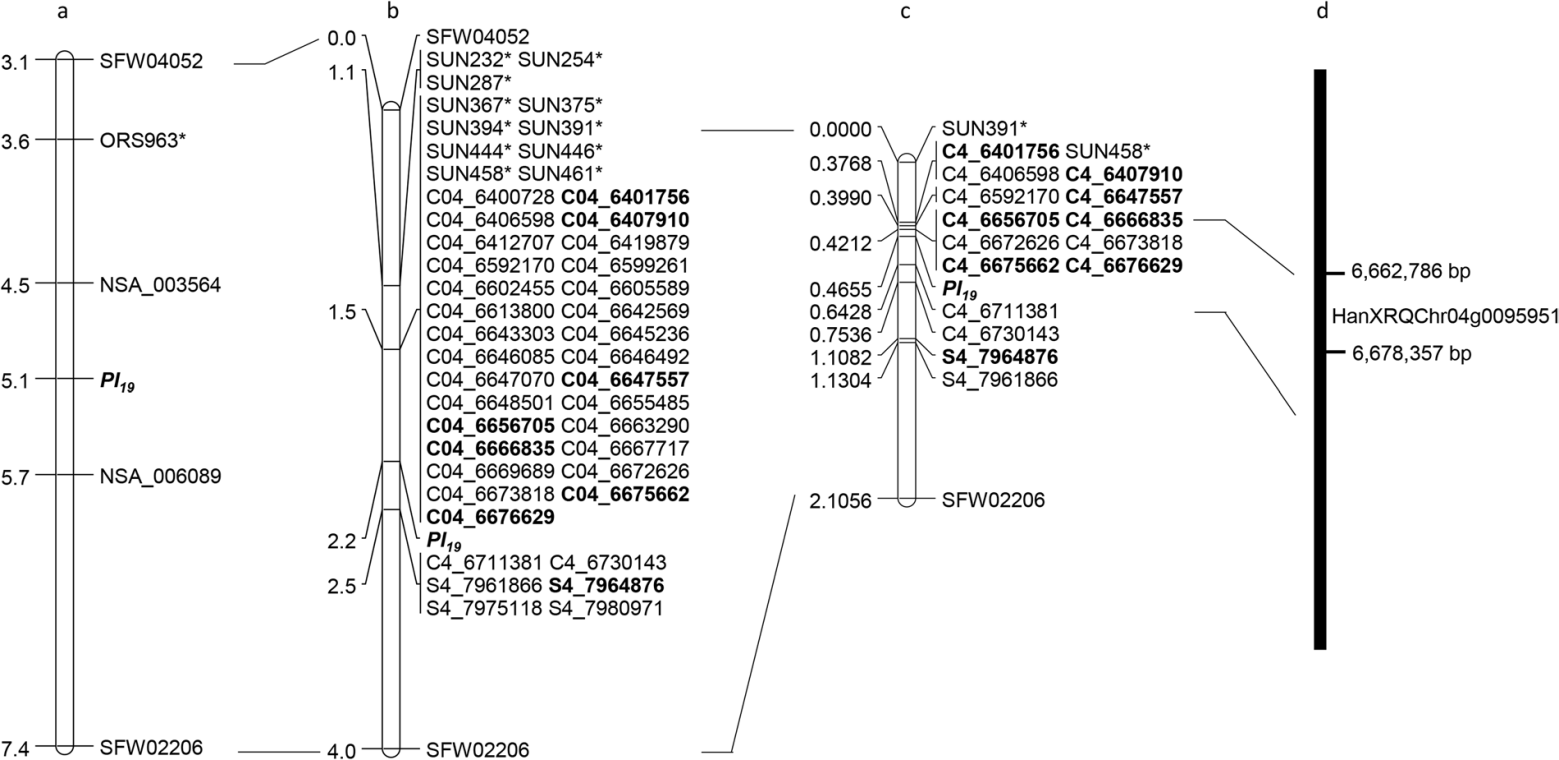
*Pl*_19_ genetic maps. (**a**) *Pl*_19_ basic map (Zhang et al. 2017); (**b**) *Pl*_19_ saturation map; (**c**) *Pl*_19_ fine map; (**d**) physical position of the *Pl*_19_ candidate gene. The diagnostic marker for *Pl*_19_ is shown in bold. *SSR/STS markers.

One hundred and one SSR and STS markers previously used in the *Pl*_17_ saturation mapping were also used to genotype the two parents of the *Pl*_19_ population, CONFSCLB1 and PI 435414. In addition, 56 SSRs were identified from the 296.4 kb sequence of XRQ from 6,238,999 to 6,535,440 bp on chromosome 4. Of 157 SSR and STS markers tested, 11 showed polymorphisms between the parents and were further used to genotype the BC_1_F_2_ population (Table 1). Linkage analysis of marker-trait associations indicated that all SSR markers mapped distal to *Pl*_19_ (Fig. 2a,b).

Based on the physical positions of the newly mapped SSR marker SUN461, which was located from 7,383,392*–*7,383,599 bp in HA412-HO and 6,413,020*–*6,413,227 bp in XRQ, 104 SNPs were selected from a 308.4 kb region (7,690,106*–*7,998,497 bp) of the HA412-HO sequence, and 168 SNPs were selected from a 398.7 kb region (6,400,728*–*6,799,385 bp) of the XRQ sequence. Of 272 SNP markers tested in CONFSCLB1 and HA-DM5, 66 were polymorphic and were used to genotype the 139 BC_1_F_2_ individuals derived from the cross of CONFSCLB1 × PI 435414 (*Pl*_19_). Total of 35 SNPs were mapped around *Pl*_19_, with four SNPs designed from the HA412-HO assembly and 31 designed from the XRQ assembly. A total of 37 co-segregating markers, including eight SSR and 29 SNP markers, were mapped to a 0.7 cM genetic distance distal to *Pl*_19_ (Fig. 2b).

To further fine map *Pl*_19_, the SSR marker SUN391 and the SNP marker SFW02206 were used as the flanking markers to screen the 2,256 BC_1_F_3_ individuals selected from the BC_1_F_3_ families heterozygous for *Pl*_19_. A total of 77 BC_1_F_3_ individuals with recombination events close to the *Pl*_19_ gene were identified and advanced to the next generation. Of 77 recombinants, 23 with recombination events occurred in the proximity to the *Pl*_19_ region, and their families (30 seedlings per family) were tested with *P*. *halstedii* race 734. Of 35 mapped SNP markers, 15 were selected for further genotyping of the 77 recombinants to increase the map resolution. The *Pl*_19_ gene was placed in the 0.2216 cM interval, flanked by SNP markers C4_6676629 (0.0443 cM) and C4_6711381 (0.1773 cM) (Fig. 2c). This genetic region corresponds to a 35 kb segment in the XRQ assembly (Table 3).

**Table 3:** Genetic and physical positions of markers linked to *Pl*_19_ on the fine map of sunflower chromosome 4.

Marker	No. recombination	Genetic distance (cM)	Physical position on XRQ assembly (bp)	Physical position on HA412-HO assembly (bp)
SUN391^†^		0	6,335,242–6,335,461	6,791,952–6,792,172
**C4_6401756**	17	0.3768	6,401,556–6,401,956	7,393,446–7,393,846
SUN458^†^	0	0.3768	6,404,243–6,404,440	7,390,959–7,391,156
C4_6406598	0	0.3768	6,406,398–6,406,798	7,556,125–7,556,525
**C4_6407910**	0	0.3768	6,407,710–6,408,110	7,557,437–7,557,837
C4_6592170	1	0.3990	6,591,970–6,592,370	7,494,498–7,494,879
**C4_6647557**	0	0.3990	6,647,357–6,647,757	7,139,515–7,139,915
**C4_6656705**	1	0.4212	6,656,505–6,656,905	6,989,404–6,989,804
**C4_6666835**	0	0.4212	6,666,635–6,667,035	6,780,829–6,781,126
C4_6672626	0	0.4212	6,672,426–6,672,826	6,975,030–6,975,273
C4_6673818	0	0.4212	6,673,618–6,674,018	6,974,002–6,974,401
**C4_6675662**	0	0.4212	6,675,462–6,675,862	6,972,167–6,972,567
**C4_6676629**	0	0.4212	6,676,429–6,676,829	6,971,201–6,971,601
*Pl*_19_	2	0.4655	—	—
C4_6711381	8	0.6428	6,711,181–6,711,581	7,089,348–7,089,748
C4_6730143	5	0.7536	6,729,943–6,730,343	7,073,422–7,073,822
**S4_7964876**	16	1.1082	6,914,409–6,914,809	7,964,676–7,965,076
S4_7961866	1	1.1304	6,911,074–6,911,439	7,961,666–7,962,066
SFW02206	44	2.1056	7,120,950–7,121,068	8,837,009–8,837,128

^†^SSR markers. The diagnostic SNP marker for *Pl*_19_ is shown in bold.

### Collinearity of SNPs between the two reference genome assemblies

In the present study, two reference genomes, HA412-HO and XRQ, were used for SNP marker development. Most SNPs from either HA412-HO or XRQ had a collinear order in both genome assemblies (Tables 2 and 3). However, of 104 SNPs selected from HA412-HO in a region of 308. 4 kb for *Pl*_19_, only four SNP markers were mapped to the *Pl*_19_ region. A search for these SNP positions in the XRQ genome assembly revealed that 30 SNPs residing in a 57.9 kb segment (7,690,106*–*7,748,049 bp) of HA412-HO were aligned to a 7.2 Mb segment (147,656,203*–*154,868,683 bp) of XRQ, which is outside the *Pl*_19_ region (Supplementary Table S3). The remaining 74 SNPs in a 236.2 kb region (7,762,321*–*7,998,497 bp) were aligned to a corresponding region of 518.1 kb (6,458,182*–*6,976,291 bp) in the XRQ assembly.

### Identification of candidate genes for Pl_17_ and Pl_19_

Most SNP markers mapped around the *Pl*_17_ locus were physically between 5,676,065 to 5,711,324 bp on chromosome 4 of the XRQ assembly (Table 2). The genetic positions of those markers were generally in accordance with their physical positions, although there was some conflict. The 104 kb genomic sequence of XRQ was analyzed from 5,670,000 to 5,780,000 bp on chromosome 4 encompassing the newly identified SNP markers from the XRQ sequence (https://www.heliagene.org/HanXRQ-SUNRISE/). Four putative genes were found in the corresponding genomic region (Table 4). One defense-associated gene HanXRQChr04g0095641 at nucleotide positions from 5,672,715 to 5,705,044 bp with a length of 32.329 kb had the typical TNL motif of the resistance gene model, encoding the full-length Toll/interleukin-1-receptor, nucleotide-binding site, and leucine-rich repeat. Moreover, all 12 polymorphic SNP markers identified from the fine mapping were in this 32.329 kb region, supporting its candidacy for *Pl*_17_ (Fig. 1d).

**Table 4:** Predicted genes in the intervals of *Pl*_17_ *and Pl*_19_ from the XRQ assembly.

Candidate gene	Description	Physical position	Length (bp)
**For** ***Pl***_***17***_			
HanXRQChr04g0095641	Probable disease resistance protein (TIR-NBS-LRR class) family, 1099 aa	5672715–5705044	32,330
HanXRQChr04g0095661	Putative protein phosphatase 2 A regulatory subunit PR55; Six-bladed beta-propeller, TolB-like	5766455–5770291	3,843
HanXRQChr04g0095671	Putative TIR domain, 217 aa	5770348–5772558	2,211
HanXRQChr04g0095681	Putative transposase (putative), gypsy type	5772609–5781483	8,875
**For** ***Pl***_***19***_			
HanXRQChr04g0095941	Putative myc-type, basic helix-loop-helix (bHLH) domain, 298 aa	6646897–6648479	1,583
HanXRQChr04g0095951	Probable RNA methyltransferase family protein, 571 aa	6662786–6678357	15,572
HanXRQChr04g0095961	Uncharacterized protein, supported by expression data, 61 aa	6730142–6755053	24,912

*Pl*_19_ was located between marker C4_6676629 and C4_6711381, and the good collinearity of the genetic and physical positions of markers in this region suggested the presence of *Pl*_19_ in the interval from 6,676,629*–*6,711,381 bp on chromosome 4 of the XRQ assembly (Table 3). A 120-kb genomic sequence on XRQ chromosome 4 was analyzed from 6,640,000 to 6,760,000 bp, which covers newly identified SNP markers for *Pl*_19_ (Table 3). Three putative genes were discovered, with one candidate gene HanXRQChr04g0095951 falling into the interval of 6,676,629*–*6,711,381 bp, which was predicted as a probable RNA methyltransferase family protein (Table 4, Fig. 2d).

### Development of diagnostic markers for Pl_17_ and Pl_19_

Currently, three different DM resistance genes, *Pl*_17_, *Pl*_19_, and *Pl*_33_, have been mapped to a similar position on sunflower chromosome 4^16,17,19^. The 12 and 35 SNP markers mapped to *Pl*_17_ and *Pl*_19_, respectively, were first tested in three resistant lines, HA 458 (*Pl*_17_), HA-DM5 (*Pl*_19_), and TX16R (*Pl*_33_), and three susceptible lines, HA 234, CONFSCLB1, and HA 434. Seven and 17 SNP markers mapped to *Pl*_17_ and *Pl*_19_, respectively, showed unique PCR pattern in HA 458 and HA-DM5 each, in contrast to the two other resistant and susceptible lines. These markers were further genotyped in an evaluation panel with 96 selected sunflower lines (Supplementary Table S4) to determine their specificity in the sunflower population and to assess their potential in marker-assisted selection for *Pl*_17_ and *Pl*_19_.

**Figure 3:**
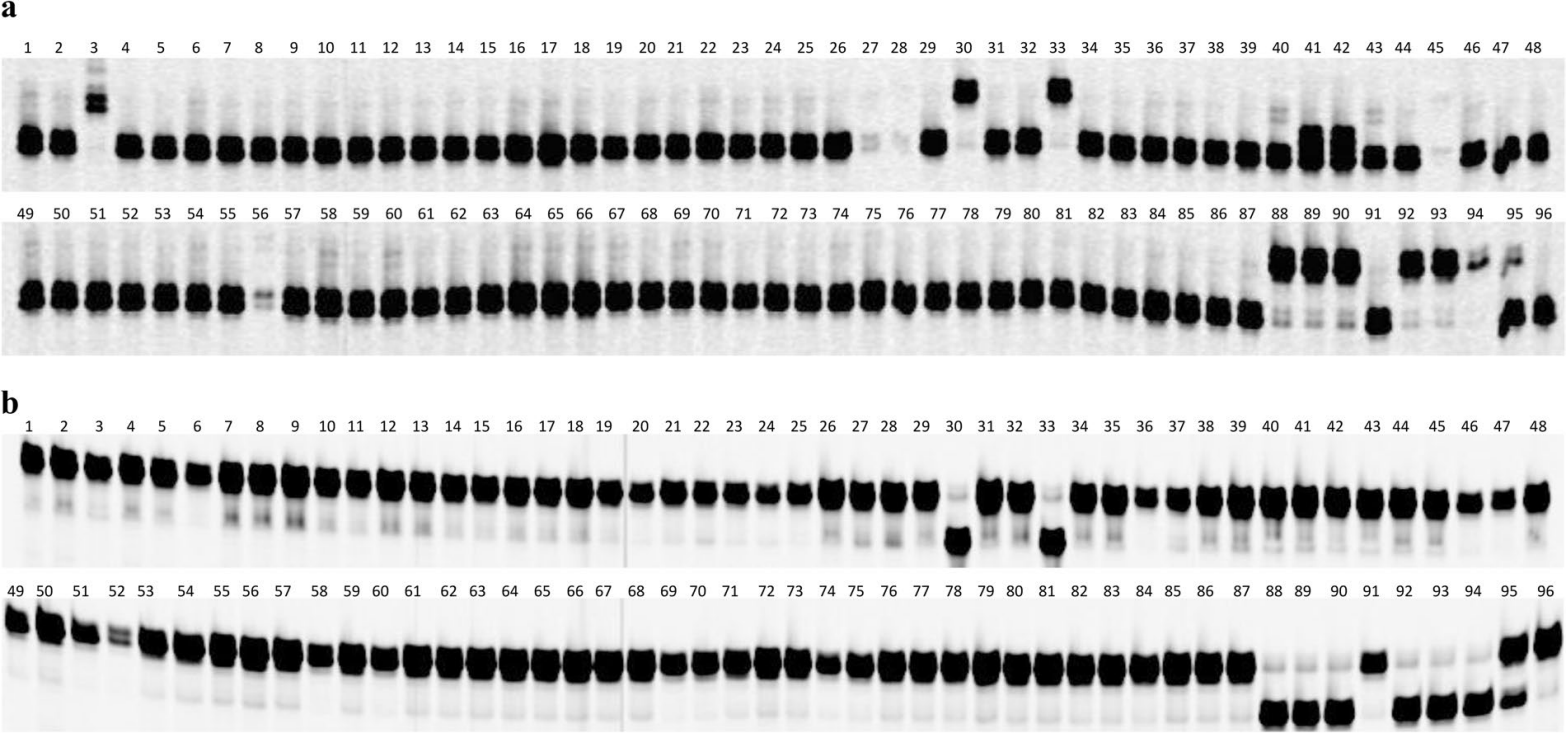
The polymerase chain reaction (PCR) amplification pattern of 96 selected sunflower lines with *Pl*_17_ diagnostic single nucleotide polymorphism (SNP) markers. (**a**) PCR amplification pattern with SNP marker C4_5696413. (**b**) PCR amplification pattern with SNP marker SPB0001. Names and pedigrees of 96 selected sunflower lines (lanes) are listed in Supplementary Table S4. Lane 30: HA 458, lane 33: HA-DM3, lane 88: HA-BSR2, lane 89: HA-BSR3, lane 90: HA-BSR4, lane 92: HA-BSR6, lane 93: HA-BSR7, and lane 94: HA-BSR8, all of which have the *Pl*_17_ gene and show the *Pl*_17_ marker allele.

Six of the seven SNP markers, C4_5696413, C4_5705018, C4_5705841, C4_5709499, SPB0001, and SPB0007, could differentiate *Pl*_17_ from other reported *Pl* genes, including *Pl*_*Arg*_, *Pl*_1_*–Pl*_3_, *Pl*_6_*–Pl*_13_, *Pl*_15_*–Pl*_21_, *Pl*_33_, and *Pl*_34_ in the selected sunflower lines (Fig. 3). HA 458 (*Pl*_17_ donor line) and those sunflower lines introgressed with the *Pl*_17_ gene, including HA-DM3, HA-BSR2 to HA-BSR4, and HA-BSR6 to BA-BSR8, showed unique *Pl*_17_ SNP marker alleles, distinguishing them from other sunflower lines (Fig. 3). The SNP marker C4_5696413 also amplified a fragment with a similar size to the *Pl*_17_ allele in HA 291 (lane 3 in Fig. 3a). Sunflower line HOLS 1 showed a heterozygous pattern in all six diagnostic SNP markers for *Pl*_17_ (lane 95 in Fig. 3).

**Figure 4:**
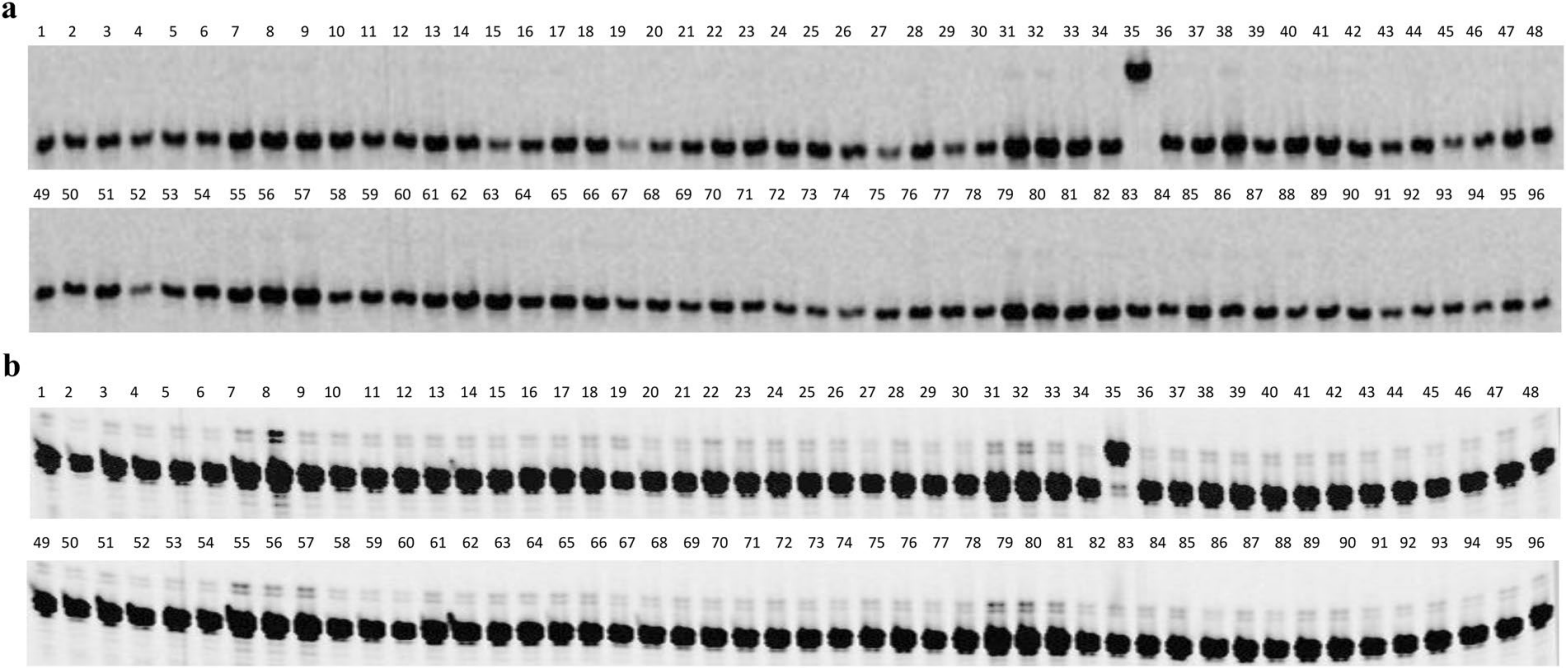
The PCR amplification pattern of 96 selected sunflower lines with *Pl*_19_ diagnostic SNP markers. (**a**) PCR amplification pattern with SNP marker C4_6666835, and b: PCR amplification pattern with SNP marker S4_7964876. Names and pedigrees of 96 selected sunflower lines (lanes) are listed in Supplementary Table S4. Lane 35: HA-DM5 with the *Pl*_19_ gene.

Of 17 SNP markers tested in the evaluation panel, eight, C4_6401756, C4_6407910, C4_6647557, C4_6656705, C4_6666835; C4_6675662, C4_6676629, and S4_7964876, could differentiate *Pl*_19_ from other reported *Pl* genes in the selected sunflower lines. HA-DM5 was the only sunflower line carrying the *Pl*_19_ gene in the 96-line evaluation panel and had a unique PCR pattern of *Pl*_19_ marker alleles compared with the remaining 95 lines (Fig. 4). These *Pl*_17_ and *Pl*_19_ unique markers are of essential utility in sunflower breeding to assist selection for these two genes.

## Discussion

Like other crops, DM *R* genes in sunflower are organized in clusters in the sunflower genome, such as in chromosomes 1 (cluster 1, *Pl*_*Arg*_, *Pl*_23_, *Pl*_24_, and *Pl*_35_; cluster 2, *Pl*_13_, *Pl*_14_, *Pl*_16_, and *Pl*_25_), 4 (*Pl*_17_, *Pl*_19_, *Pl*_27_*–Pl*_29_, and *Pl*_33_), 8 (*Pl*_1_, *Pl*_2_, *Pl*_6_, *Pl*_7_, *Pl*_15_, and *Pl*_20_), and 13 (*Pl*_5_, *Pl*_8_, *Pl*_21_, *Pl*_22_, *Pl*_31_, *Pl*_32_, and *Pl*_34_) (Supplementary Table S1)^23–28^. Distinguishing genes from a cluster can be achieved through traditional allelic analysis, polymorphic marker analysis, resistance specificity to different pathotypes, and the presence or absence of host reactions to pathogen effectors. Our previous studies have indicated that *Pl*_17_ and *Pl*_19_ are different but closely linked genes on sunflower chromosome 4 (data for allelic analysis not shown). Common markers ORS963 and NSA_003564 are downstream of *Pl*_17_ but upstream of *Pl*_19_^16,17^. Both genes are delimited in an interval of 3.2 Mb on chromosome 4 of the HA412-HO assembly, at which time the XRQ reference was not available. Using a sequence-based chromosome walking strategy toward the target gene in this study, *Pl*_17_ was refined into an interval of 15 kb at a position from 5,696,076*–*5,711,324 bp on chromosome 4 in the XRQ assembly. In contrast, *Pl*_19_ was precisely mapped to an interval of 35 kb at a position from 6,676,429*–*6,711,781 bp in the XRQ assembly, approximately 1 Mb apart from *Pl*_17_. A recently reported DM *R* gene, *Pl*_33_, is located in an interval of 1.56 Mb from 4,208,180*–*5,766,419 bp on chromosome 4 in the XRQ assembly^19^. Marker analysis among the three gene donors suggested that *Pl*_33_ is different from *Pl*_17_ and *Pl*_19_ (Figs 3 and 4).

Pecrix *et al*. (2018) reported mapping of the three new DM *R* genes, *Pl*_27_*–Pl*_29_, to the upper end of sunflower chromosome 4, a similar region to that of *Pl*_17_ and *Pl*_19_^18^. Both *Pl*_27_ and *Pl*_28_ originate from a wild perennial sunflower *H*. *tomentosus* (synonym *H*. *tuberrosus*), while *Pl*_29_ is derived from the same source as *Pl*_17_, *Pl*_19_, and *Pl*_33_ of the wild annual sunflower *H*. *annuus*. The *Pl*_17_ donor accession PI 468435 was collected from Idaho, USA, and both accessions of PI 435414 for *Pl*_19_ and Texas-16 for *Pl*_33_ were collected in Texas, USA, while *Pl*_29_ accession Wyoming 358 was collected from Wyoming, USA^16–19^. The flanking markers place *Pl*_27_ in an interval between 2.18*–*6.40 Mb, *Pl*_28_ between 6.62*–*8.42 Mb, and *Pl*_29_ between 6.93*–*7.07 Mb in the XRQ assembly, respectively^18^. Three genes fall within a small region between *Pl*_17_ and *Pl*_19_ on chromosome 4 with *Pl*_29_ close to *Pl*_19_. Further fine mapping of *Pl*_27_*–Pl*_29_ or cloning of *Pl*_17_ and *Pl*_19_ will elucidate the genetic architecture and evolutionary mechanisms underlying this gene cluster.

The sunflower genome is approximately 3.6 Gb in size with more than 80% highly repetitive sequences. The assembly of a large and complex genome with a high level of repetitive sequences remains a challenge in the community, but longer read length, higher genome coverage, and more sophisticated bioinformatics would reduce this difficulty and provide more accurate results. The HA412-HO whole-genome sequence was assembled from Illumina reads (100 bp) and 454 Roche reads (400*–*1,000 bp), while the XRQ whole-genome sequence was assembled from PacBio sequencing data with an average read length of 10.3 kb^20^. High quality genome assembly is crucial for reference sequence-based chromosome walking to anchor a specific region for the target gene. In the present study, comparison of mapped SNP positions between two assemblies revealed the coincidence of their positions in the two reference genomes of most SNPs. However, when searching the positions of 104 SNPs derived from HA412-HO for *Pl*_19_ in the XRQ genome assembly, 30 SNPs located in a 57.9 kb segment between 7,690,106 and 7,748,049 bp were found to align to a 7.2 Mb segment between 147,656,203 and 154,868,683 bp in XRQ (Supplementary Table S3), and none of them was mapped to the *Pl*_19_ region. This finding complicates the use of the reference genome for chromosome walking.

The two sunflower reference sequences provide alternative opportunities for SNP discovery. In the current study, SNPs from the XRQ genome showed more polymorphisms than those from HA412-HO. A total of 120 SNPs from HA412-HO were used for *Pl*_17_ (16 SNPs) and *Pl*_19_ (104 SNPs) fine mapping, and only four were mapped (3.3%). In contrast, of 232 SNPs from XRQ tested for *Pl*_17_ (64 SNPs) and *Pl*_19_ (168 SNPs), 43 were mapped (18.5%). Considering its assembly from very long PacBio reads, the XRQ genome sequence can be used as the first choice in sequence-based chromosome walking aiming for fine mapping and gene cloning in the sunflower community, while the HA412-HO genome provides a useful comparison to the XRQ genome and a second selection of SNP markers.

In the prior five years, 18 new DM *R* genes (*Pl*_17_*–Pl*_20_, *Pl*_22_*–Pl*_35_) have been identified and mapped with a total of 36 DM *R* genes in sunflower^1,16–19,29–32^. Despite this great progress, none of the DM *R* genes has been cloned in sunflower to date. The *R* genes cloned from other crops indicate that most *R* genes encode proteins with nucleotide binding and leucine-rich repeat domains (NLRs)^33,34^. The putative candidate gene HanXRQChr04g0095641 identified from the reference genome of XRQ for *Pl*_17_ belongs to this class. A preliminary expression analysis suggested it is potentially a *Pl*_17_ gene with the expected kinetics in cotyledons and roots between susceptible and resistant parents in chronological order (data not shown). EMS-induced mutation was performed in a large population of HA 458 seeds and advanced into the M_2_ generation. DM testing of the M_2_ population is currently underway to screen for mutants showing susceptible phenotypes. The sequences of the candidate gene HanXRQChr04g0095641 will be further evaluated and compared between wild type and mutants. These studies will provide a foundation to facilitate our efforts of cloning *Pl*_17_ in the future.

The 35 kb region of the XRQ reference genome harboring *Pl*_19_ contains only one annotated gene, HanXRQChr04g0095951, predicted as a probable RNA methyltransferase family protein. RNA methylation and its role in human diseases have been reported, however, genes with similar annotation have not thus far been implicated in disease resistance in plants^35,36^. Genomic regions harboring plant disease resistance genes are often complex, exhibiting structural variations between resistant and susceptible genotypes^37^. Thus, it is possible that the *Pl*_19_ gene is absent from the available sunflower reference assemblies. Alternatively, the single gene identified at the *Pl*_19_ locus in the XRQ assembly may be indicative of a novel resistance mechanism. Similarly, although a more conventional prospective candidate gene was identified for *Pl*_17_, the gene conferring resistance may also be absent from the reference assembly. Future work on the cloning of *Pl*_17_ and *Pl*_19_ will be required to distinguish between these possibilities and elucidate the genetic basis of the broad-spectrum disease resistance.

Downy mildew remains the major disease threat to sunflower production because of its high-level ability to develop new virulence and its worldwide distribution. Two prerequisites are essential to the use of host resistance in breeding programs, i.e., a resistance resource and diagnostic markers. Both *Pl*_17_ and *Pl*_19_ show broad-spectrum resistance to all known isolates of *P*. *halstedii*^10,17,18^. Because of their biallelic nature, SNP markers show fewer polymorphisms in the breeding population in nature, especially if the marker is not closely linked to the target gene. In the current study, we applied a whole-genome resequencing approach combined with reference sequence-based chromosome walking to narrow down the gene intervals and develop diagnostic SNP markers for *Pl*_17_ and *Pl*_19_, respectively. Six diagnostic SNP markers for *Pl*_17_ spanned a physical distance of 15 kb in the XRQ genome within the candidate gene HanXRQChr04g0095641. Two diagnostic SNP markers, C4_6675662 and C4_6672629, closest to *Pl*_19_ were in a 35-kb interval of *Pl*_19_ within the candidate gene HanXRQChr04g0095951. The high-density maps and diagnostic SNP markers for *Pl*_17_ and *Pl*_19_ developed in this study provide useful tools to accelerate the transfer of these genes to elite sunflower lines in breeding programs, as well as facilitate pyramiding of these genes with other broadly effective *Pl* genes for durable DM control^38^.

## Methods

### Mapping populations and evaluation panel

The initial F_2_ mapping population for *Pl*_17_ was created from a cross between HA 234 and HA 458 with 186 individuals. HA 458 (PI 655009) is an oilseed maintainer line that is resistant to all North American *P*. *halstedii* races identified thus far. HA 234 is an oilseed sunflower maintainer line that is susceptible to DM. The DM resistance gene *Pl*_17_ in HA 458 was previously mapped to sunflower chromosome 4^16^. This F_2_ population was used for saturation mapping of additional markers in the present study. For fine mapping, recombinants were screened from 3,008 F_3_ individuals selected from the previously characterized F_2:3_ families heterozygous for *Pl*_17_. Each selected heterozygous F_3_ family equates to a segregating F_2_ population.

Saturation mapping of the DM *R* gene *Pl*_19_ was performed in the BC_1_F_2_ population developed from the cross of cytoplasmic male sterile (CMS) CONFSCLB1 and PI 435414 with 139 F_2_ individuals, which was previously used for the initial mapping of *Pl*_19_^17^. PI 435414, which is resistant to DM, is a wild *H*. *annuus* accession that was collected from Paris, Texas, U.S. in 1978. CONFSCLB1 is a confectionary maintainer line that is susceptible to DM. For fine mapping, recombinants were screened from 2,256 BC_1_F_3_ individuals selected from the previously characterized BC_1_F_2:3_ families heterozygous for *Pl*_19_. In our follow-up breeding program, *Pl*_19_ was successfully introgressed from wild PI 435414 into confectionary sunflower, named HA-DM5 (PI 687025), which was used for whole-genome resequencing to fine map the *Pl*_19_ gene.

The evaluation panel consisted of 96 sunflower inbred lines with diverse origins, including 24 and 17 lines harboring different DM and rust *R* genes, respectively (Supplementary Table S4). This panel was used to identify diagnostic DNA markers in marker-assisted selection for *Pl*_17_ and *Pl*_19_, respectively.

### SSR and STS marker identification

Previous genetic mapping studies have placed both *Pl*_17_ and *Pl*_19_ on sunflower chromosome 4 in an interval between 3,621,089 and 6,852,749 bp^16,17^. This stretch of 3.2 Mb genomic sequence covering both loci was extracted from the HA412-HO (https://www.heliagene.org/HA412.v1.1.bronze.20141015/) and XRQ reference genomes (GenBank accession GCA_002127325.1), respectively. The type and distribution of simple sequence repeats (SSRs) were analyzed using GRAMENE Ssrtool (http://archive.gramene.org/db/markers/ssrtool), and those repeated no less than five times were utilized for primer design. Sequence-tagged sites (STSs) were also analyzed within this 3.2 Mb sequence of the HA412-HO reference. A total of 157 pairs of primers, including 40 STSs and 117 SSRs (40 STSs and 55 SSRs were from the HA412-HO sequence and 62 SSRs from the XRQ sequence), were designed for amplification.

### Resequencing and SNP marker identification

Initially, HA 458 whole genome sequence (named HA458-WGS1 with a low genome coverage) was provided by Dr. Loren Rieseberg of the University of British Columbia, Canada, and aligned with the reference genome XRQ (https://www.heliagene.org/HanXRQ-SUNRISE/) around the *Pl*_17_ region to identify SNPs and InDels. Subsequently, HA 458 (HA458-WGS2) and HA-DM5 (released germplasm with *Pl*_19_) were sequenced at 40 and 35 × depth, respectively, on the Illumina HiSeq sequencing platform at CD Genomics Inc. according to their protocols. Briefly, quality DNA samples were used for library construction using CoVaris S/E210 for fragmentation, and qualified libraries for each gene were pooled for sequencing. Raw reads resulting from sequencing process were filtered to remove reads containing adaptors, reads with >1% ambiguous bases, and reads with low quality (greater than 50% bases less than 15 Q score). A total of 961,980,260 (98.95%) clean reads were obtained from HA 458 sequencing, where 952,508,154 (99.02% mapping rate) and 954,743,565 (99.25%) reads could be mapped to the HA412-HO and XRQ reference genomes, respectively. All SNPs and InDels were identified using the mapped reads and annotated with ANNOVAR software. HA-DM5 was also whole-genome resequenced at CD Genomics Inc. with the same protocols and on the same platforms. The SNP markers were named with prefix C4 or S4 followed by a number representing the physical position of the SNPs along chromosome 4 of each reference genome assembly. C4 represents the SNP from the XRQ reference, while prefix S4 represents the SNP from the HA412-HO reference.

### Genotyping of PCR-based markers and linkage analysis

SSR and STS primers were designed using the Primer 3 program (Table 1)^39,40^. For SNP genotyping, primers were designed as described by Qi *et al*. (2015)^16^ and Long *et al*.^21^ based on SNP flanking sequences (Supplementary Tables S2, S5). Polymerase chain reaction (PCR) for SSR and STS was performed as described by Qi *et al*. (2011)^41^, while SNP PCR was conducted as described by Qi *et al*. (2016)^32^. PCR products were visualized by gel electrophoresis on a 6.5% polyacrylamide gel using an IR2 4300/4200 DNA analyzer (LI-COR, Lincoln, NE, USA).

Genotyping data for each marker was first assessed for goodness of fit to the Mendelian segregation ratio (1:3 for dominant and 1:2:1 for codominant) using the Chi-square (χ^2^) test. Those fitted markers were linkage analyzed with phenotyping data using JoinMap 4.1 software^42^. Regression mapping algorithm and Kosambi’s mapping function were chosen. The cutoffs of linkage analysis among markers were set at a likelihood of odds (LOD) ≥3.0 and maximum genetic distance ≤50 centimorgans (cM).

### Downy mildew resistance evaluation

The *P*. *halstedii* isolate of race 734 was chosen to test seedlings of the recombinants selected from the fine mapping populations for resistance to DM, together with their respective parents, HA 234 and HA 458 for *Pl*_17_, and CONFSCLB1 and HA-DM5 for *Pl*_19_, using the whole seedling immersion method as described by Gulya *et al*.^43^ and Qi *et al*. (2015)^16^. Race 734 was first identified in 2009 in North America and overcame the *Pl*_6_ and *Pl*_7_ genes^8^. The seedling was considered susceptible (S) if sporulation was observed on cotyledons and true leaves and resistant (R) if no sporulation was observed. A total of approximately 30 seedlings from each recombinant were inoculated with the *P*. *halstedii* isolate of race 734 and evaluated. The recombinants were classified as homozygous resistant if none of the seedlings exhibited sporulation, segregating if some seedlings showed sporulation on cotyledons and true leaves, and homozygous susceptible if all seedlings showed sporulation on cotyledons and true leaves, which represented the genotypes of DM resistance in each recombinant.

### Ethical standards

The experiments were performed in compliance with the current laws of the USA.

## Supplementary information

Supplementary Tables S1

Supplementary Tables S2

Supplementary Tables S3

Supplementary Tables S4

Supplementary Tables S5
